# Machine learning classifies predictive kinematic features in a mouse model of neurodegeneration

**DOI:** 10.1038/s41598-021-82694-3

**Published:** 2021-02-17

**Authors:** Ruyi Huang, Ali A. Nikooyan, Bo Xu, M. Selvan Joseph, Hamidreza Ghasemi Damavandi, Nathan von Trotha, Lilian Li, Ashok Bhattarai, Deeba Zadeh, Yeji Seo, Xingquan Liu, Patrick A. Truong, Edward H. Koo, J. C. Leiter, Daniel C. Lu

**Affiliations:** 1 Department of Neurosurgery, David Geffen School of Medicine, University of California, Los Angeles, 300 Stein Plaza, Ste. 536, Los Angeles, CA 90095-6901 USA; 2 Neuromotor Recovery and Rehabilitation Center, David Geffen School of Medicine, University of California, Los Angeles, Los Angeles, CA 90095 USA; 3 Brain Research Institute, University of California, Los Angeles, Los Angeles, CA 90095 USA; 4 Department of Kinesiology, Nutritional and Food Sciences, California State University, Los Angeles, Los Angeles, CA 90032 USA; 5 Office of Knowledge Enterprise Development, Arizona State University, Tempe, AZ 85281 USA; 6 College of Osteopathic Medicine, Touro University Nevada, Henderson, NV 89014 USA; 7 The Ohio State University Wexner Medical Center, Columbus, OH 43210 USA; 8 Department of Neuroscience, San Diego School of Medicine, University of California, San Diego, La Jolla, CA 92093 USA; 9 Department of Molecular and Systems Biology, Geisel School of Medicine of Dartmouth College, Lebanon, NH 03756 USA; 10 School of Information, University of California, Berkeley, Berkeley, CA 94720 USA

**Keywords:** Dynamical systems, Learning algorithms, Motor control, Alzheimer's disease, Computational biology and bioinformatics, Neurology, Signs and symptoms

## Abstract

Motor deficits are observed in Alzheimer’s disease (AD) prior to the appearance of cognitive symptoms. To investigate the role of amyloid proteins in gait disturbances, we characterized locomotion in APP-overexpressing transgenic J20 mice. We used three-dimensional motion capture to characterize quadrupedal locomotion on a treadmill in J20 and wild-type mice. Sixteen J20 mice and fifteen wild-type mice were studied at two ages (4- and 13-month). A random forest (RF) classification algorithm discriminated between the genotypes within each age group using a leave-one-out cross-validation. The balanced accuracy of the RF classification was 92.3 ± 5.2% and 93.3 ± 4.5% as well as False Negative Rate (FNR) of 0.0 ± 0.0% and 0.0 ± 0.0% for the 4-month and 13-month groups, respectively. Feature ranking algorithms identified kinematic features that when considered simultaneously, achieved high genotype classification accuracy. The identified features demonstrated an age-specific kinematic profile of the impact of APP-overexpression. Trunk tilt and unstable hip movement patterns were important in classifying the 4-month J20 mice, whereas patterns of shoulder and iliac crest movement were critical for classifying 13-month J20 mice. Examining multiple kinematic features of gait simultaneously could also be developed to classify motor disorders in humans.

## Introduction

Alzheimer’s disease (AD) has a long preclinical stage, during which its characteristic pathologies accumulate, even as they are insufficient to confirm a clinical diagnosis based on the onset of cognitive impairment. Although motor function was previously thought to be spared until later stages of AD, there is increasing evidence that motor deficits are present in preclinical AD^1^. In particular, gait dysfunction progresses^2,3^ as the degree of cognitive impairment worsens^4^, and early motor deficits may contribute to an increased risk of falling^5^. Gait is a complex movement requiring coordination of multiple body parts to produce a stereotypical motor pattern^6–11^. The synergy among multiple body parts is disrupted by AD^12–18^. Patients with mild AD display slower gait velocities, longer stance times, shorter step lengths, and compromised stepping posture stability^16^. However, current analytical techniques cannot associate gait pattern changes with a specific pathology because neuropathological changes at any level of the sensorimotor system generating locomotion can be reflected in an abnormal gait, and thus, gait disorders are diagnostically non-specific^19,20^.

Three-dimensional kinematic analysis of gait has emerged as a powerful tool for quantitative assessment in subjects with a wide range of neurological conditions^20–26^. It provides information about trajectories, velocities, accelerations, and angles of movement of different body parts. It is an ideal choice for assessing movement in patients: it is non-invasive^27^; it allows repeated assessment within a short period of time^28^; and it provides quantitative and comprehensive kinematic data. Automatic^28,29^ or semi-automatic^30^ kinematic analysis systems are available and have been used extensively in diagnosing and evaluating various interventions in patients with severe motor deficits and distinct gait patterns from known causes^26,31,32^, such as spinal cord injury (SCI)^33–35^ and Parkinson’s Disease (PD)^7,29,36,37^.

Despite the opportunity to extract and quantify a variety of gait features, three-dimensional kinematic analysis is applicable mainly in pathological conditions with a known kinematic pattern constructed from a pre-selected feature set^38–40^. Current motion capture software provides built-in kinematic analysis, but only compares a limited number of kinematic variables that demonstrate distinct differences between pathological and control groups^23,39,41^. Although quantitative measurements of gait have been developed^19,42–44^ to assist in rehabilitation^34,45^ and to evaluate the effectiveness of various therapies^25^, quantitative gait assessment as a diagnostic tool is in its infancy because representative kinematic features of specific pathologies remain uncharacterized^7,46–48^.

**Figure 1:**
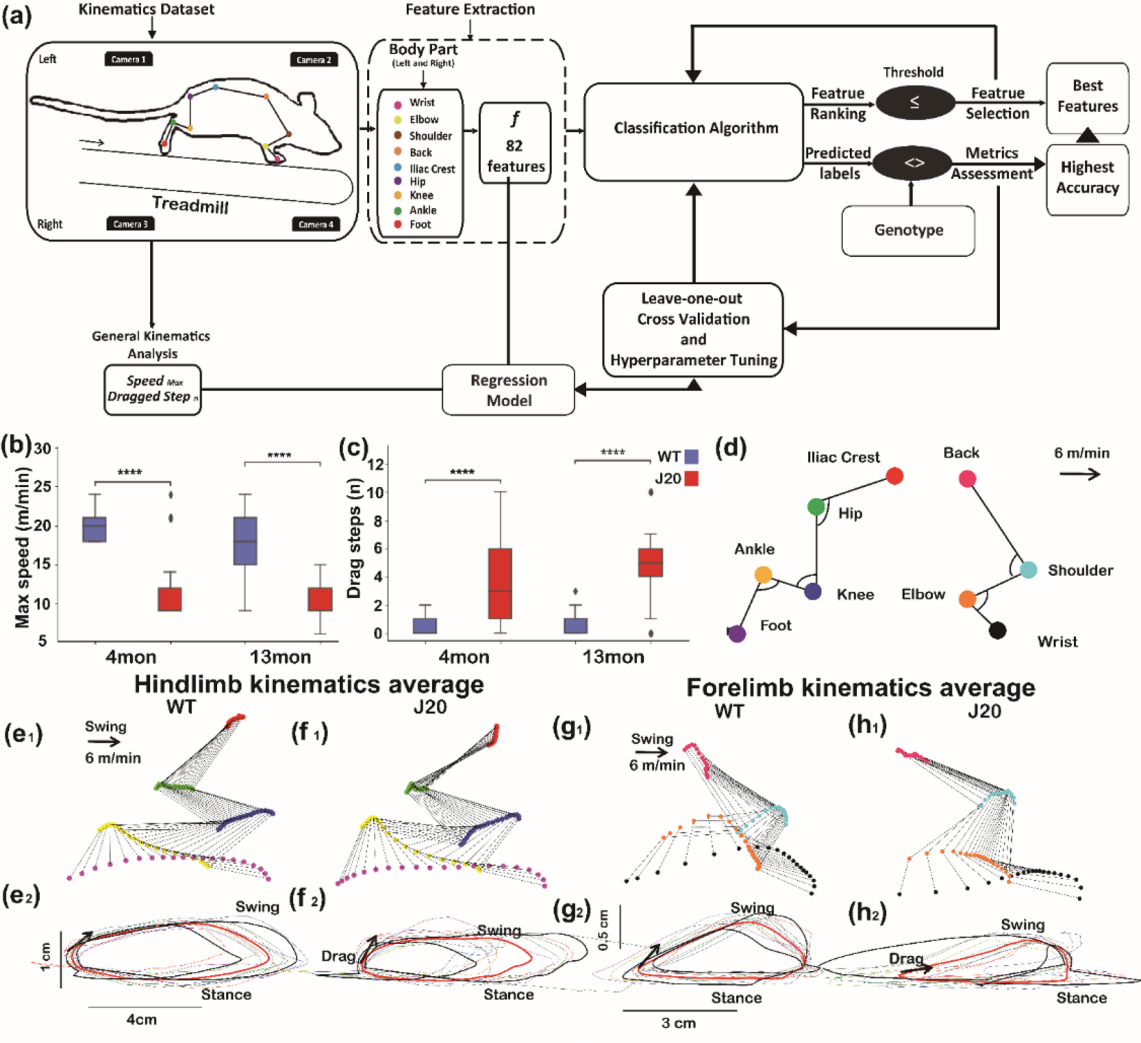
A comparison of kinematic features of locomotion between J20 and wild-type mice. (**a**) Schematic of analytical methods used. Kinematic features were extracted from nine anatomical locations and subsets of the features were optimized for genotype classification of sixteen transgenic mice and fifteen wild-type mice. Regression models were also established to determine the subsets of features predictive of the maximum speed or the drag step number. Kinematic analysis of locomotion revealed a difference in the mean maximum speed (**b**) and mean number of dragged steps (**c**) between J20 and WT mice during locomotion. The mice were stratified into two age-groups: 4-month-old mice (4mon) and 13-month-old mice (13mon; *: P < 0.05, **: P < 0.01, ***: P < 0.0001). Typical angular relationships of the forelimb and hindlimb of mice are shown (**c**). Averaged trajectories of the hindlimbs of WT (**e**) and J20 (**f**) mice as well as the forelimbs of WT (**g**) and J20 (**h**) mice are shown during quadrupedal locomotion using the color scheme for each joint shown in (**d**). Panels generated through Python 3.7, MATLAB 9.2, Power Point (Microsoft office 2016) and Adobe Illustrator (2019).

Studies investigating gait dysfunction in mild AD have been limited to two-dimensional evaluations that do not account for the trajectories of individual body parts in space^42,49–53^. These analyses may insufficiently detect and quantify small gait changes that distinguish preclinical AD from other disorders. Furthermore, the characterization of gait in healthy subjects or experimental animals has been inadequate to establish useful diagnostic criteria for AD^51,54–58^. We asked, therefore, whether we could characterize healthy gait and gait deficits associated with AD to diagnose the disease earlier. To answer this question, we are developing sensitive computational methods to detect mild gait changes seen in preclinical AD. Toward that goal, we examined the three-dimensional kinematic patterns of gait in a well-characterized human amyloid precursor protein (hAPP) overexpressing transgenic mouse line, J20 mice, where age-associated amyloid deposits and amyloid-related pathology are detected in the brain ^59–61^. In order to study the progression of motor dysfunction in these animals, we divided animals into 4-month old (4mon) and 13-month old (13mon) groups. Amyloid deposition is progressive in J20 mice: amyloid β (Aβ) plaques are not prominent until age 5–7 months and are widespread by age 8–10 months^62^. Therefore, 4mon J20 mice model an early AD stage in that memory and learning deficits in the J20 animal are not as prominent as those in older animals^59,63–65^. The 13mon J20 mice show abundant amyloid deposits and other associated changes and manifest overt memory and learning deficits. We used machine learning to establish algorithms that identified kinematic features of gait that were unique to J20 mice and that further distinguished J20 from wild-type (WT) mice in both age groups (Fig. 1a).

## Methods

### Animal groups

All animal studies were performed according to protocols approved by the University of California, Los Angeles Animal Research Committee. The methods were carried out in accordance with the relevant guidelines and regulations as well as in full compliance of the ARRIVE (Animal Research: Reporting of in vivo Experiments) guidelines 2.0.

The J20 transgenic mouse line expresses a mutated human amyloid precursor protein (APP, K670/M671L and V717F) under control of the platelet-derived growth factor promoter (PDGF)^66^. We used a J20 mouse line bred on a C57 black 6 background. Our dataset was derived from kinematic measurements of fifteen 4mon animals (7 WT, 8 J20) and sixteen 13mon animals (8 WT, 8 J20).

### Experimental procedure for kinematic measurements

All animals were tested on a treadmill^67–70^. Reflective markers were placed bilaterally on the wrist, shoulder, thoracic spine, iliac crest, hip, knee, ankle, and foot of each animal. Video recordings were made at 100 Hz using a four-camera SIMI 2D/3D motion capture software (SIMI Reality Motion System, Unterschleissheim, Bayern). In order to obtain stable and representative gait patterns, we began with a treadmill speed of 3 m/min. This speed was increased to a maximum of 33 m/min in increments of 3 m/min once each animal was able to complete at least 10 consecutive steps at a given speed. We recorded the maximum stable running speed in addition to the number of steps with drag, defined as the incidence of toe dragging during initiation of the swing phase of each step. For each gait cycle, 800 measurements of the 3D-coordinates (*x, y, z*) of the limbs were obtained from which 82 kinematic features were derived. The 3D-coordinates (*x, y, z*) of all the tracked body parts were extracted by the SIMI software for analysis and computation.

### Feature extraction

To evaluate locomotion, we considered three important qualities: (1) the path of each body part throughout the stepping cycle; (2) the synergy among different body parts during locomotion; and (3) the patterns indicating stability of locomotion compared to normal gait^71–76^. Based on these qualities, trajectory-based and angle-based analyses were applied to extract kinematic features (Table 1). In the trajectory-based analysis, we focused on features related to movement of individual body parts during each gait cycle (Table 1, Supplementary Figure 2). In the angle-based analysis, we focused on features related to angle changes that reflected synergy among at least three body parts (Table 1, Supplementary Figure 2). Angle changes were derived from the joints labeled with reflective markers and calculated bilaterally. The markers at the iliac crest and thoracic spine were used to indicate the angular relationship between the trunk and the treadmill plane. For each step of the gait cycle measured in each animal, two bilateral instances related to the right and left side of the body were included (BL dataset; Supplementary Material), and balance of the animal during locomotion was inferred from a comparison of the contribution of features collected from the left and right body parts. Patterns indicating stability of locomotion were derived from the standard deviation of each feature analyzed, where a greater standard deviation indicated less consistent and less stable locomotion. We extracted trajectory and angle changes from at least three consecutive steps in each animal to analyze the sequence of limb motions that propelled the body forward and maintained stance stability. These pre-selected features were previously identified as crucial for assessing the movements necessary for locomotion^37,76–83^. In total, 82 features were identified and used for further analysis (Fig. 1a, Table. 1).

**Table 1:** Kinematic measurements and their definitions**.**

Feature name	Symbol	Feature definition
Step length	${sl, sl}_{BP}^{i}$	$\underset{1\le j\le N}{\mathrm{max}}\left({x}_{j,BP}^{i}\right)-\underset{1\le j\le N}{\mathrm{min}}({x}_{j,BP}^{i})$
Step height	${sh, sh}_{BP}^{i}$	$\underset{1\le j\le N}{\mathrm{max}}\left({y}_{j,BP}^{i}\right)-\underset{1\le j\le N}{\mathrm{min}}({y}_{j,BP}^{i})$
Length/height ratio	${rho, \rho }_{BP}^{i}$	${sl}_{BP}^{i}/{sh}_{BP}^{i}$
Trajectory path length	$L, {L}_{BP}^{i}$	${\sum }_{j=1}^{N}{l}_{j,BP}^{i}$
Average line velocity	${v, v}_{BP}^{i}$	$\frac{{L}_{BP}^{i}}{N}$
Maximum angle	${theta\_max, \theta }_{max, BP}^{i}$	$\underset{1\le j\le N}{\mathrm{max}}\left({\theta }_{j,BP}^{i}\right)$
Minimum angle	$theta\_min, {\theta }_{min, BP}^{i}$	$\underset{1\le j\le N}{\mathrm{min}}({\theta }_{j,BP}^{i})$
Angle average	$theta\_avg, {\theta }_{avg, BP}^{i}$	$\frac{{\sum }_{j=1}^{N}{\theta }_{j,BP}^{i}}{N}$
Angle standard deviation	$theta\_std, {\theta }_{std, BP}^{i}$	$\frac{{\sum }_{j=1}^{N}{(\theta }_{j,BP}^{i}-{\theta }_{avg,BP}^{i})}{N}$
Maximum angular speed	$angv\_max, {r}_{max,BP}^{i}$	$\underset{1\le j\le N}{\mathrm{max}}\left({r}_{j,BP}^{i}\right)$
Minimum angular speed	$angv\_min, {r}_{min, BP}^{i}$	$\underset{1\le j\le N}{\mathrm{min}}({r}_{j,BP}^{i})$
Angular speed average	$angv\_avg, {r}_{avg, BP}^{i}$	$\frac{{\sum }_{j=1}^{N}{r}_{j,BP}^{i}}{N}$
Angular speed standard deviation	$angv\_std, {r}_{std, BP}^{i}$	$\frac{{\sum }_{j=1}^{N}{(r}_{j,BP}^{i}-{r}_{avg,BP}^{i})}{N}$

Kinematic measurements were extracted bilaterally for all observed joints, except that the angular features of the foot and wrist were not extracted since the angular relations of these anatomical parts to the next distal limb elements are not easily measured and not easily related to locomotion. The variable $N$ represents the number of data points sampled in this study and equaled 800.

### Data pre-processing

MATLAB software (R2017a, MATLAB 9.2, MATLAB & Simulink, MathWorks Inc., CA, USA) was used to extract and calculate the kinematic features from the (*x, y, z*) coordinates captured with the SIMI 2D/3D software. The Scikit-learn 0.21.3 package for Python programming language was used for all pre-processing of the data and feature selection as well as for machine language model training and cross-validation.

Because amyloid proteins accumulate in animal models of AD and in humans with AD, we considered age as a potential confounding factor. To remove potential confounding by age, we trained and validated binary classification models separately on the 4mon and 13mon datasets. Consequently, all data points measured on each subject in each age group were labeled as wild type (WT) or J20 (Supplementary dataset 1). Within each group, we also modeled the unilateral movements (i.e. right or left side) to assess dependency of the results on the kinematics of either side of the body.

### Model training, testing, and evaluation

- Classifying each age group into J20 and wild type animals

Random forests (RF) were selected as the primary algorithm for classification of the ensemble datasets. We selected this algorithm for three reasons: first, it is able to rank different features in the modeling process based on their importance; second, this algorithm is fairly robust to overfitting since a large number of trees can be trained independently (and yet efficiently), and the final verdict will be based on the majority vote among the outcomes of all trees; and third, RF are an appropriate choice for small input datasets as was the case in this study. Since multiple data points were obtained for each subject in this study, a leave-one-out cross validation (LOO-CV) was used to assess the performance of the model and to prevent any leakage of data from training into the validation process. During LOO-CV, all data points measured on a particular subject were set aside for testing while the data from all other subjects were used in the model training process. This process was repeated for all subjects within each group (4mon/13mon), and the average performance across all runs is reported. For each LOO-CV run, the training and validation sets were normalized using a Min–Max normalization criterion. In an attempt to prevent overfitting and improve the model performance, Gaussian noise was added to the normalized training data in each LOO-CV run. The value for the mean and standard deviation of the Gaussian noise were tuned for each dataset to yield the highest performance in terms of the balanced accuracy.

We tried multiple classification algorithms: k-nearest neighbor (KNN), radius neighbor (RN), binomial naïve bayes (NB), logistic regression (LR), and support vector classifier (SVC), since we had paired outputs based on the same data, and we compared the difference between the pairs of model outputs using McNemar’s test^84^. The paired comparisons were performed separately within each age group between the RF and the other algorithms. For each pair of comparisons, a contingency table was prepared by counting the number of correct and incorrect predictions for each model, and McNemar’s test statistics (χ^2^) was calculated as follows:

1 $${\chi }^{2}=\frac{{\left(b-c\right)}^{2}}{b+c}$$

where *b*, *c* are the off-diagonal elements of the contingency square matrix defining the number of test instances that first model got correct and second got incorrect (Yes–No) versus the count of test instances that the first model got incorrect but the second one got right (No–Yes), respectively. The null-hypothesis for this test was defined as no difference between the two classifiers in terms of χ^2^. The threshold for statistical significance was set at 0.05. The results of these comparisons showed a p-value much less than 0.05 for all paired comparisons within each group except between RF and SVC, indicating that the RF model was superior to most models and not different from the SVC output. Although we were not able to reject the null hypothesis for the RF-SVC comparison (p > 0.05), the number of test instances that RF got correct and SVC got incorrect (Yes–No) was greater than the count of test instances that RF got incorrect and SVC got correct (No–Yes). Therefore, a RF algorithm for classification provided slightly greater accuracy than the SVC, and McNemar’s Test revealed that the RF approach had advantages over the other algorithms examined.

Metrics used to evaluate performance of the RF classification model included accuracy, balanced accuracy, F1-score, and false negative rate (or miss rate). Balanced accuracy adjusts the “regular” accuracy for unequal numbers of instances in each class by balancing between the sensitivity (the percentage of all true-positives) and the specificity (the percentage of all true-negatives)^85^. The F1-score is a measure of the balance between sensitivity and precision (positive predictive value). The miss rate (FNR) was selected considering that false-negatives (i.e. wrongly classified J20 mice as WT) would be riskier as compared to false-positives in this study in which the positive (1) and negative (0) classes are defined as J20 and WT, respectively.

- Predicting maximum speed and/or number of dragged steps

In order to evaluate the possibility of predicting the maximum speed and/or number of dragged steps, we fit a regression model to the data in each age group with both genotypes combined. Different regression models (including RF regressor, Lasso, Ridge, and the Support Vector Regressor (SVR)) were tested, and the lowest mean absolute error (MAE) and the lowest root mean squared error (RMSE) were selected as metrics to evaluate performance of the predictive model. Within each age group, the dependent variable for each regression model included either the drag step (a value between 0 and 10) or max speed (a value between 6 and 21 m/min). A leave-one-out approach for cross-validation was applied within each age group.

### Bias-variance tradeoff and hyperparameter tuning

Model overfitting was a major challenge to overcome with LOO-CV. Thus, we focused on tuning the hyperparameters that can act as regularizers that limit model complexity and mitigate the variance between model predictions derived from the training and test datasets (overfitting). To this end, the number of trees in the forest (nT), maximum depth of the trees (maxD), minimum number of samples required to split an internal node (minS), and minimum number of samples required to be at a leaf node (minSL) were selected as part of the hyperparameter tuning process. We selected the grid search method for hyper-parameter tuning, in which a grid of parameter values were exhaustively explored to find the model prediction with the optimal bias-variance tradeoff^86^.

### Feature selection

We used impurity-based feature importance^87^ embedded in the RF algorithm within the Scikit-learn package as the primary algorithm to define feature importance for all attributes in the modeling process. A general concern when using impurity-based feature importance methods is that the method may introduce bias based on unusual or unique values or values that are outliers (i.e., values with high cardinality). To address this, we used an alternative approach called permutation feature importance^88^. In this method, the increase in the model prediction error after permuting the values of a feature is used as a measure of the importance of that feature. In other words, the more sensitive the model is to random shuffling of feature values, the more important the feature is in the modelling process. Thus, there should be no bias with this method because the distribution of variables is preserved. Our metric to select the most appropriate algorithm for feature selection was based on the balanced accuracy of the retrained models using the features selected by each method.

By setting a threshold for feature importance within each method, we were able to select features that made the largest contribution identifying the genotype of the animals. In order to assess the effectiveness of feature selection methods, we used reduced datasets obtained from the selected features to retrain and retest all classification algorithms. The threshold for feature importance was optimized to achieve the least number of selected features that also yielded comparable or greater accuracy during the second stage modeling with a reduced feature set as compared to use of the full datasets.

In contrast to the impurity-based approach that is native to the RF model, the permutation importance can be used with any model, and thus it was used to assess the feature ranking in the SVR modeling. The scoring metric for the permutation importance in the SVR model was selected to minimize RMSE regression loss.

### Statistics

The variables are presented as the mean ± the standard deviation (SD). All the statistical analyses were conducted with R (3.3.6). When individual variables were analyzed, a univariate mixed effects model was first used to analyze each kinematic variable as a function of genotype as the prediction outcome (**g**, Eq. 2). The kinematic variable (***F***_***n***_, Eq. 2) was treated as the fixed factor while ID of the animal (***ID***, Eq. 2) and the drag step (***DS***) from which the features were extracted were treated as random factors. The univariate mixed effect model equation was formulated as follows:

2 $$\mathbf{g}={{\varvec{F}}}_{{\varvec{n}}}{\varvec{\beta}}+({\varvec{I}}{\varvec{D}}/{\varvec{D}}{\varvec{S}}){\varvec{u}}+{\varvec{\varepsilon}}$$

where ***β*** and ***u*** are the coefficients and ***ε*** is the error term. The function summary (lm.n) was used to get the p-value of the models based on asymptotic Wald test^89^. A one-way ANOVA was performed to evaluate differences among models by examining the mean squares associated with each model. Each univariate mixed effect model was also evaluated by their Akaike (AIC), Bayesian information criteria (BIC) and log-likelihood. The statistics presented in Fig. 1a were based on this one-way ANOVA testing, and the p-value was calculated based on the Bonferroni-Sidak post-hoc test. Only those variables with a P-value ≤ 0.05 were considered statistically significant.

## Results

### J20 mice demonstrated similar gait deficits to patients with AD

Slow stepping speed and elevated fall risk are two important features of gait dysfunction in patients with advanced AD, and we compared maximum stepping speeds and number of steps with drag (definition in supplementary Figure 1) between J20 and WT mice (Fig. 1b, c). Analysis of maximum locomotory speed revealed substantial effects of genotype (F = 54.5; p < 0.0001 5.49, 1 degrees of freedom; df); the average maximum running speed was consistently slower in J20 mice compared to WT mice. Additionally, both strains ran marginally slower at 13 months compared to 4 months (F = 4.63; p = 0.031 and 1 df; Fig. 1b). All animals were capable of sustained locomotion at a speed of 6 m/min; therefore, the number of dragged steps among 10 consecutive steps was counted for all the animals at this speed. Within both the 4mon and 13mon groups, we observed dragged steps more frequently in J20 than in WT mice (4mon p = 0.00094, 13mon p < 0.0001). The number of dragged steps did not differ significantly according to age in either the J20 or WT groups (WT p = 3.016, J20 p = 0.3187) (Fig. 1c).

The sequential angular relationships of the hind- and forelimbs are shown in Fig. 1e1, f1, g1 and h1, and the averaged trajectories of the joints in the 4mon dataset are shown in Fig. 1e2, f2, g2 and h2. Note that the joint trajectories are smaller and more variable in the 4mon J20 mice, indicating that the locomotory coordination among different parts of each limb was less in the 4mon WT mice.

### Random Forest algorithm correctly classified the animals into genotype groups

To test whether the kinematic pattern differences at running speeds less than 6 m/min could be used to classify animals into their correct genetic groups, we applied a supervised random forest classification algorithm after tuning the hyper-parameters to achieve the best model performance. The LOO-CV balanced accuracy of the RF model was 92.3 ± 5.2% using the 4mon BL dataset, 88.5 ± 8.3% with the 4mon right-side-only dataset, 96.2 ± 3.8% with the 4mon left-side-only dataset, 93.3 ± 4.5% using the 13mon BL dataset, 96.6 ± 3.3% with the 13mon right-side-only dataset, 93.3 ± 4.5% with the 13mon left-side-only dataset (Table 2). Moreover, the miss rate (false negative rate) was zero for all model outcomes but the 4mon right-side-only dataset (Table 2). High values of the F1-score (Table 2) also indicated a healthy balance between sensitivity and precision. Thus, analysis of kinematic features during locomotion at a comfortable speed using machine learning can distinguish the AD-model, J20 genotype from WT mice, before (4mon) and after (13mon) the onset of typical memory and learning deficits.

**Table 2:** Evaluation of different classification algorithms.

Data	Hyper-parameter values	Acc mean ± SEM (%)	B-Acc mean ± SEM (%)	F_1_-score mean ± SEM (%)	FNR mean ± SEM (%)
4mon BL	nT = 500, maxD = 6, minS = 0.00001, minSL = 5	96.2 ± 3.1	92.3 ± 5.2	97.7 ± 1.9	0.0 ± 0.0
4mon R	nT = 500, maxD = 8, minS = 0.00001, minSL = 5	86.9 ± 9.0	88.5 ± 8.3	88.2 ± 8.4	7.7 ± 7.7
4mon L	nT = 500, maxD = 5, minS = 0.00001, minSL = 7	93.0 ± 6.9	96.2 ± 3.8	93.7 ± 6.3	0.0 ± 0.0
13mon BL	nT = 500, maxD = 4, minS = 0.00001, minSL = 3	99.3 ± 0.5	93.3 ± 4.5	99.6 ± 0.2	0.0 ± 0.0
13mon R	nT = 500, maxD = 4, minS = 0.00001, minSL = 3	99.3 ± 0.6	96.6 ± 3.3	99.6 ± 0.3	0.0 ± 0.0
13mon L	nT = 500, maxD = 4, minS = 0.00001, minSL = 3	98.0 ± 1.4	93.3 ± 4.5	98.9 ± 0.8	0.0 ± 0.0

Mean and standard error of the mean (SEM) of all leave-one-out cross validation classification accuracies (Acc), balanced accuracies (B-Acc), F1-Score, and False Negative Rate (FNR). Data were derived from the bilateral (BL), right-side-only (R), left-side-only (L), within each 4-months-only (4mon), and 13-month-only (13mon) datasets. Definition of the RF model hyper-parameters: RF-nT: number of trees, maxD: minimum depth of the tree, minS: minimum samples required to split an internal node, minSL:

### RF model with the impurity-based feature selection method yield the best resolution of feature ranking

The random forest was retrained using reduced datasets identified using feature selection methods for each age group to determine which selected features were sufficient to classify animal genotype correctly. Model training and cross validation algorithms were repeated three times using the following reduced datasets for each age group to produce feature importance (FI) subsets:

1. features selected by impurity-based method (FI_IMP_).
2. features selected by permutation method (FI_PER_).
3. common features between impurity-based and permutation methods (FI_IMP∩PER_).

We determined the thresholds for feature selection in the BL dataset in each age group that produced both the least number of features and the greatest accuracy in the retrained model. Using the default RF feature and both impurity-based and permutation feature importance models resulted in 22 selected features (Supplementary Figure 2e,f). Similar accuracy and miss rates were observed with reduced datasets (Supplementary Table 1 and Supplementary Figure 2)*.* Feature importance for the remaining attributes varied as a function of the feature-ranking method, the classification-training dataset, or both. However, the permutation-based feature selection did not separate the features as cleanly as the impurity-based feature selection methods (Supplementary Figure 2e,f). Of the 22 highest-ranked features, the top 16 and the top 12 features were common among the two feature selection methods for the 4mon and 13mon groups, respectively, which confirms that the impurity-based method is fairly unbiased, at least for those features that make a strong contribution in the modeling process. Moreover, the impurity-based method requires fewer steps in the modeling process (i.e. embedded in the RF modeling process) and also has better scalability to larger datasets. Thus, we built our feature ranking based on the RF model with the impurity-based feature selection for all subsequent analyses of feature importance (Table 1).

### The importance of the features for genotype classification differed between 4-month and 13-month age groups

We compared the top 22 important features for genotype classification in 4mon and 13mon groups. For the 4mon group, the average of angle velocity of hip (*HP_angv_mean*) ,the average of angle range of elbow(EL_theta_ravg), the minimum angle of shoulder (*SH_theta_min*), the minimum and standard deviation of hip angle (*HP_theta_min, HP_theta_std*) were the top five features. For the 13mon group, length-to-height ratio of shoulder kinematics (*SH_rho*), the length of iliac crest movement (IL_C_l), the average of angle range of hip (*HP_theta_ravg*), the mean velocity of foot and ankle (*FT_v_mean, AK_v_mean*) were the top five features.

**Figure 2:**
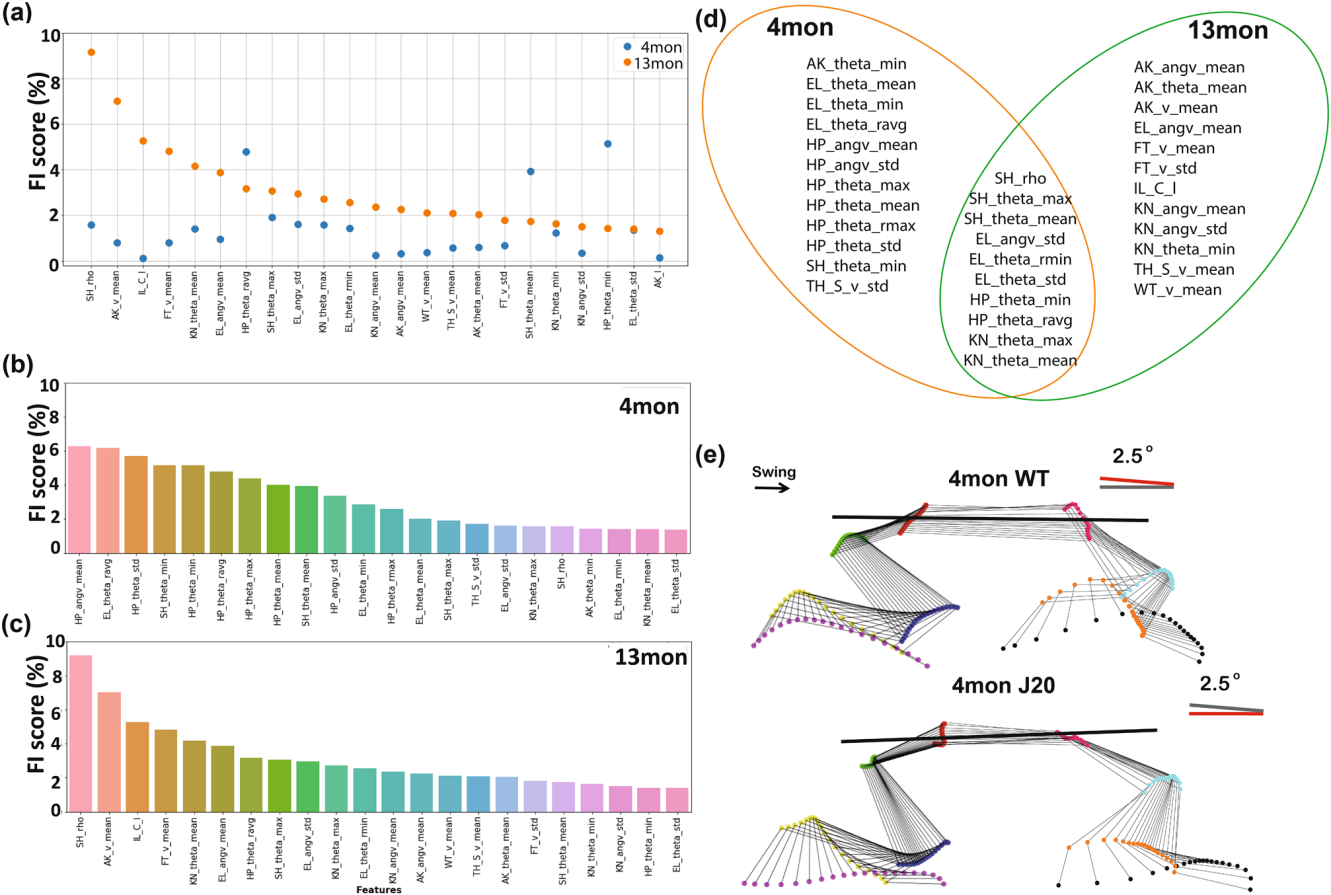
Feature importance (FI) scores (%) revealed age-specific kinematic deficits in 4mon and 13mon J20 mice. (**a**) A summary of FI scores (%) from the 4-month-bilateral (4mon-BL), and 13-month-bilateral (13mon-BL) datasets; (**b**) ranking of 4mon-BL FI scores (%); and (**c**) ranking of 13mon-BL FI scores (%). (**d**) A diagram of the 22 highest-ranked features of the 4mon, 13mon, and full bilateral datasets circled in orange, green, and blue, respectively. (**e**) A 2.5-degree tilt of the trunk was observed in the 4mon J20 when compared to their littermates in the WT group. Thus, subtle differences between genotypes could be detected even from data acquired at 4 months of age. Panels generated through Python 3.7, MATLAB 9.2, and Adobe Illustrator (2019).

The top ranking features of 4mon and 13mon were very different, especially the top 10 features between the two age groups (Fig. 2b, c). Among the 22 highest-ranked features, 10 features were common for the 4mon and 13mon groups, while the other 12 features for each group were unique to each age group (Fig. 2d). For example, the most important feature for genotype classification in the 13mon mice, length-to-height ratio of shoulder kinematics (sh_*ρ*), was not an important feature distinguishing the J20 from WT groups using the 4mon dataset (Fig. 2a–c). While the 22 highest-ranked features of 13-month group were distributed among all nine tracked joints, the high-ranked features of 4-month group aggregated among elbow, shoulder, hip, and knee. Among these four joints, hip features contributed the most to genotype classification for the 4-month group, 8 out of 22 features were related to the hip (Fig. 2b–d). Whereas features from extremities (wrist, foot, and ankle) were more important for genotype classification of the 13mon (Fig. 2d).

The features important for genotype classification in 4mon mice were more related to angular features; the 14 highest-ranked features of the 4mon dataset were all angular features (Fig. 2c, d). These angular features are related to changes in posture, we compared the coordinates of the thoracic spine (*x, y, z*) and iliac crest (*x, y, z*) to the treadmill plane. A 2.5-degree increase in trunk and pelvic axis angulation was detected between the 4mon WT and 4mon J20 mice (Fig. 2e). Because the angular features were calculated based on the kinematics of the observed joints and two of its adjacent joints (supplementary Figure 1), these findings indicate that the synergistic relationship of adjacent joints was more affected in the 4mon J20 mice than the 13mon J20 mice. Thus, hAPP-overexpression seemed to alter the kinematics of locomotion differently at the two ages examined.

### 13mon group demonstrated a more imbalanced left–right kinematics pattern compared to 4mon group

**Figure 3:**
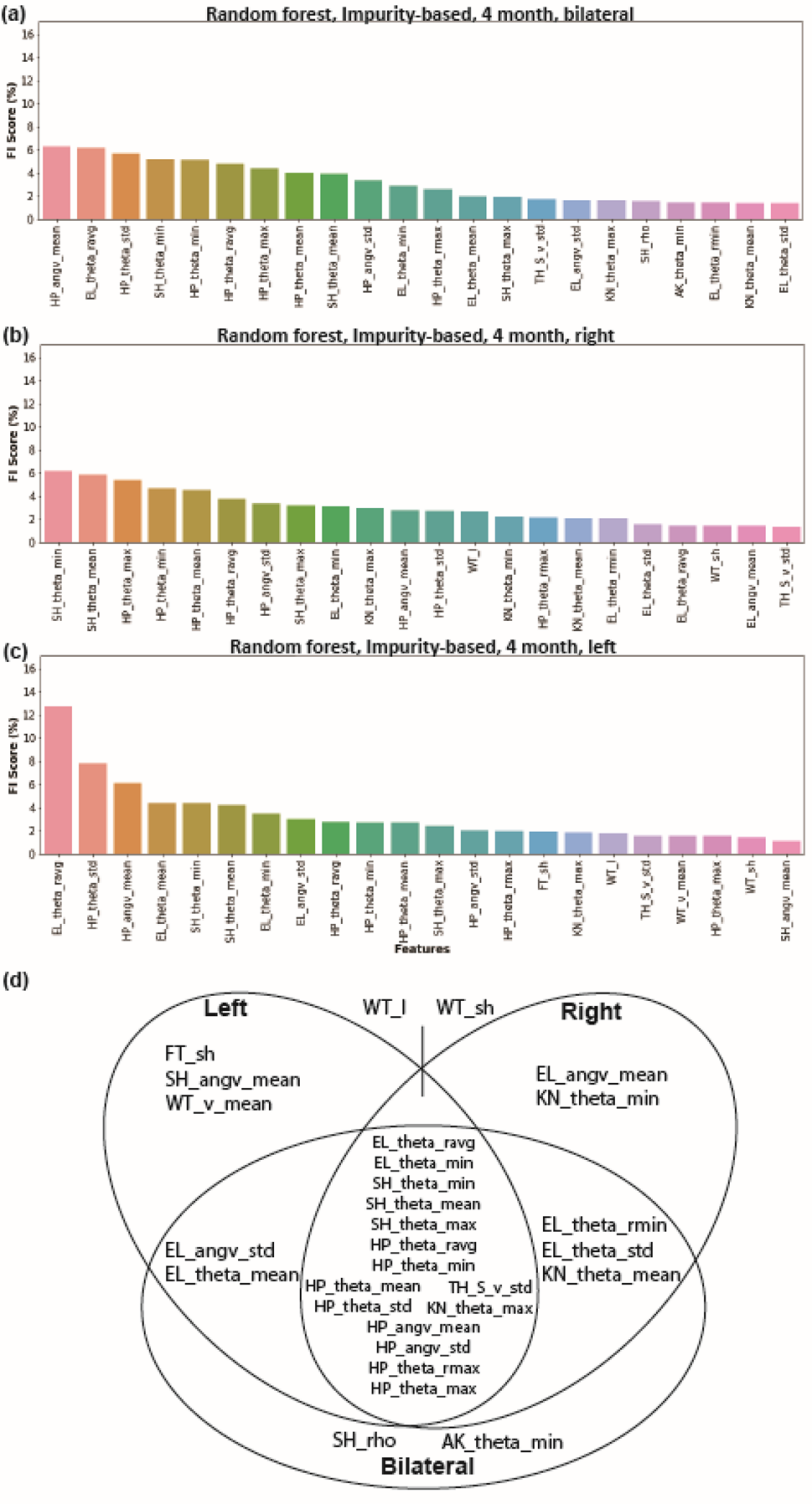
Left- and right-side-only datasets yield different rankings of feature importance score, while still sharing the majority features in the 4mon J20 mice. Bilateral and unilateral Feature Importance (FI) scores (%) calculated by the Random Forest feature importance method using the bilateral (**a**), right-side-only (**b**), and left-side-only (**c**) datasets. The 22 highest-ranked features, which made the largest contribution towards identifying genotype and age are shown. (**d**) A summary diagram of the 22 highest-ranked features of the left-side-only, right-side-only, and bilateral datasets indicating their common and unique features. Please see the feature definition equations for abbreviations used for each feature (Table 1). Panels generated through Python 3.7, MATLAB 9.2, and Adobe Illustrator (2019).

**Figure 4:**
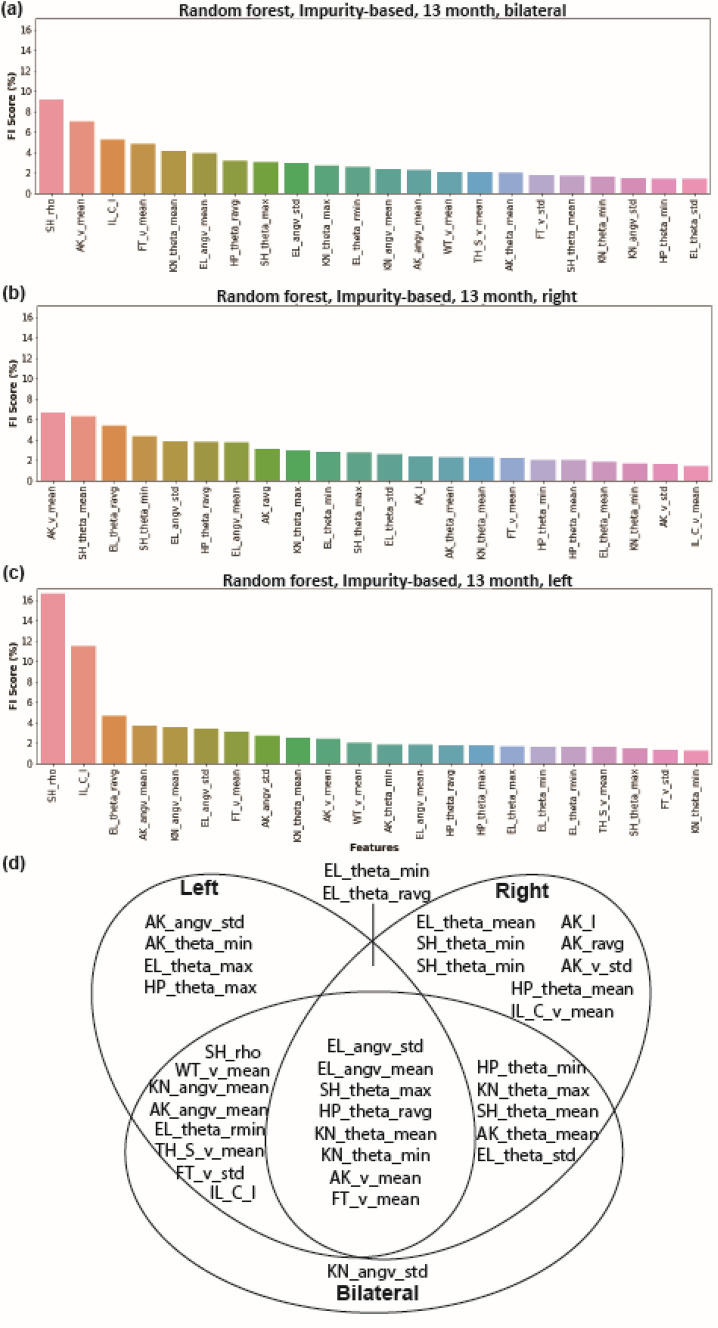
Left- and right-side-only datasets yield different rankings of feature importance score, with fewer shared features in the 13-mon J20 mice. Bilateral and unilateral Feature Importance (FI) scores (%) calculated by the Random Forest feature importance method using the bilateral (**a**), right-side-only (**b**), and left-side-only (**c**) datasets. The 22 highest-ranked features, which made the largest contribution towards identifying genotype and age are shown. (**d**) A summary diagram of the 22 highest-ranked features of the left-side-only, right-side-only, and bilateral datasets indicating their common and unique features. Please see the feature definition equations for abbreviations used for each feature. Panels generated through Python 3.7 and Adobe Illustrator (2019).

Loss of balance during stepping is one of the major motor deficits of patients with AD, and we investigated whether left–right imbalance is observed in the J20 mice when compared to WT. We performed additional genotype classification in each age group based on left-side-only (4mon left only, 13mon left only) and right-side-only (4mon right only, 13mon right only). The 22 highest-ranked features for genotype classification selected based on bilateral, right and left dataset from 4mon group (Fig. 3) and 13mon group (Fig. 4) were compared to determine if there existed a difference between the feature set from each side of the animal. For the 4mon group, there were 15 features out of 22 common among bilateral, left and right group. The left and right of 4mon shared 17 out 22 features (Fig. 3). There were only 10 out of 22 common features between the 13mon-left and 13mon-right (Fig. 4). Notably, the most important feature, *SH_rho*, in the 13mon-bilateral and 13mon-left datasets was not among the top 22 features of the classification based on 13mon-right dataset, indicating a strong difference between the kinematic features collected from left and right side of the animal.

### The cumulative FI scores (%) from each body part elucidated J20-specific gait pattern

**Figure 5:**
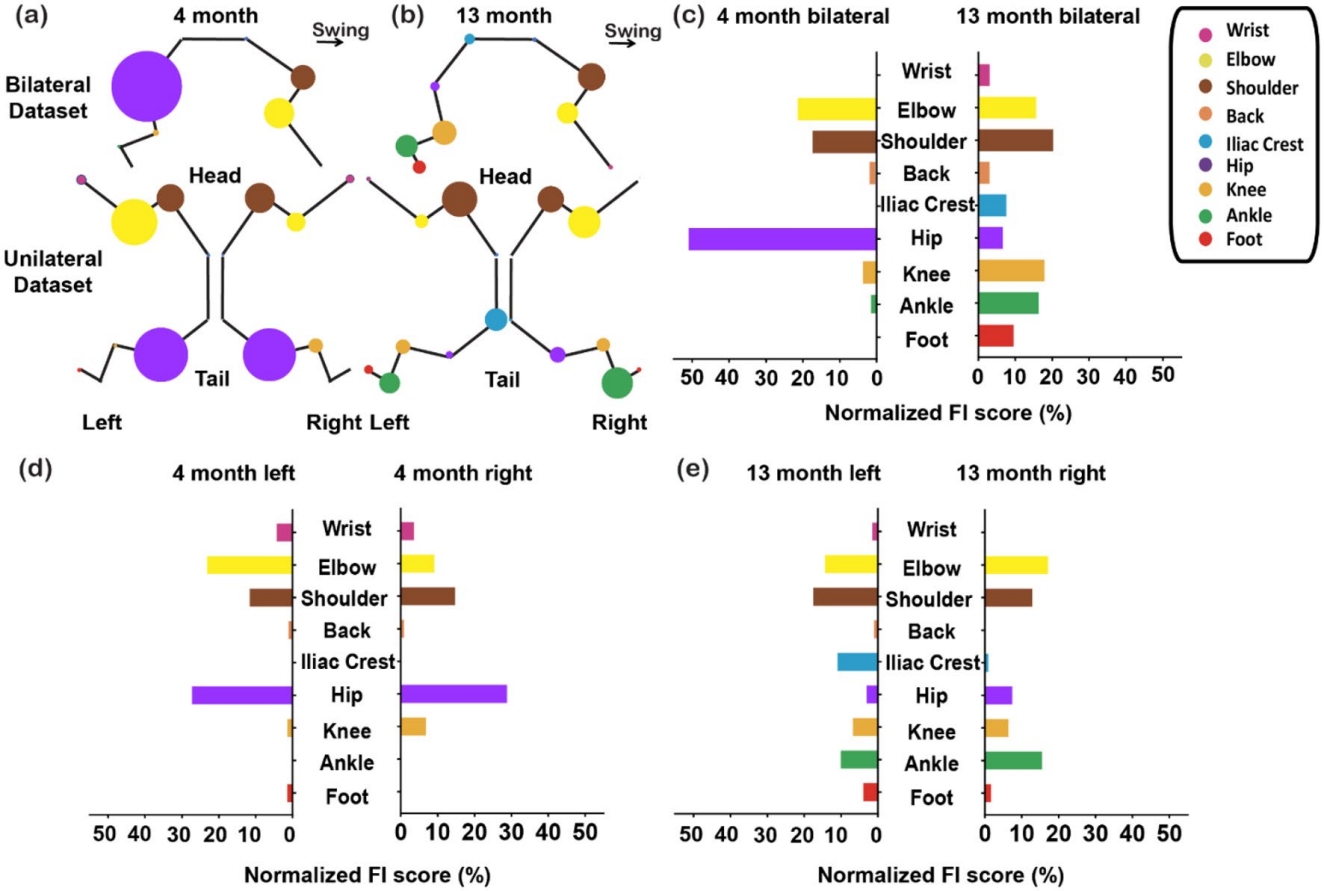
Feature scores for each anatomical location in different datasets. Four-month-only and 13mon-only datasets yielded different cumulative FI scores (%) for different anatomical locations, suggesting that kinematic synergy (or the lack thereof) during locomotion was correlated with age and pathological progression. Each bubble shown represents an anatomical location analyzed in this study. The size of the bubble is proportionate to its cumulative FI score calculated from bilateral or unilateral datasets of (**a**) the 4mon-only group, and (**b**) the 13mon-only group. The cumulative FI scores (%) were calculated by summing all the FI scores related to each anatomical location. All 82 features extracted from all body parts were included. As the mice aged, more joints become important for differentiating the two genotypes instead of just the hip, shoulder, and elbow (**c**). The left–right comparison was also performed for 4mon (**d**) and 13mon (**e**) respectively. Panels generated through MATLAB 9.2 and Adobe Illustrator (2019).

We calculated the contribution of each body part to the observed gait pattern by calculating the cumulative FI scores (%) generated by the RF model using the 4mon and 13mon datasets (Supplementary Table 2). The contributive weight of each body part was also calculated using FI scores separately from BL and unilateral (L, R) datasets (Fig. 5a, b) from the two age groups. The cumulative FI scores was highest for the hip in the 4mon bilateral group. The importance of the hip dramatically declined in the 13mon group. The importance of the elbow also dropped slightly when all the FI scores of other joints increased in the 13mon bilateral group (Fig. 5c). In a comparison of FI scores between the 4mon-left and 4mon-right data, the left elbow had a higher FI score than the right, and the right knee had a higher FI score than the left. The FI scores of the other joints were similar between the two sides (Fig. 5d). Interestingly, features from the wrist were important based on left or right only datasets in the 4mon group, but yielded a 0 FI score with the 4mon-bilateral dataset when the bilateral data pooled together (Fig. 3d, 5a&d, Supplementary Table 2). This suggested that either motor differences existed in unilateral wrist movements, which canceled or compensated for each other when combined in the bilateral dataset (a biological effect), or the variance in the feature values within the combined bilateral dataset was diluted in a way that resulted in smaller relative number of splits across all trees that include the feature it that ultimately precluded detection of each unilateral effect (a mathematical effect inherent in the analysis).

The left–right differences of shoulder, iliac crest, hip, ankle and foot FI scores were detected in the 13mon animals (Fig. 5e). The biggest FI score difference was between the left and right iliac crest (left: 1.44%, right: 11.48%, difference: 10.04%) followed by the difference between left and right ankle (left: 16.01%, right: 10.61%, difference: 5.39%) and shoulder (left: 13.42%, right: 18.04%, difference: 4.58%).

In summary, the motion of hip, the balance between left and right elbow and knee were most affected in the younger group of J20 mice, while abnormal kinematics of the lower extremities and imbalance of lower trunk movement were more prominent in older J20 mice.

### Drag step and maximum speed analysis

**Figure 6:**
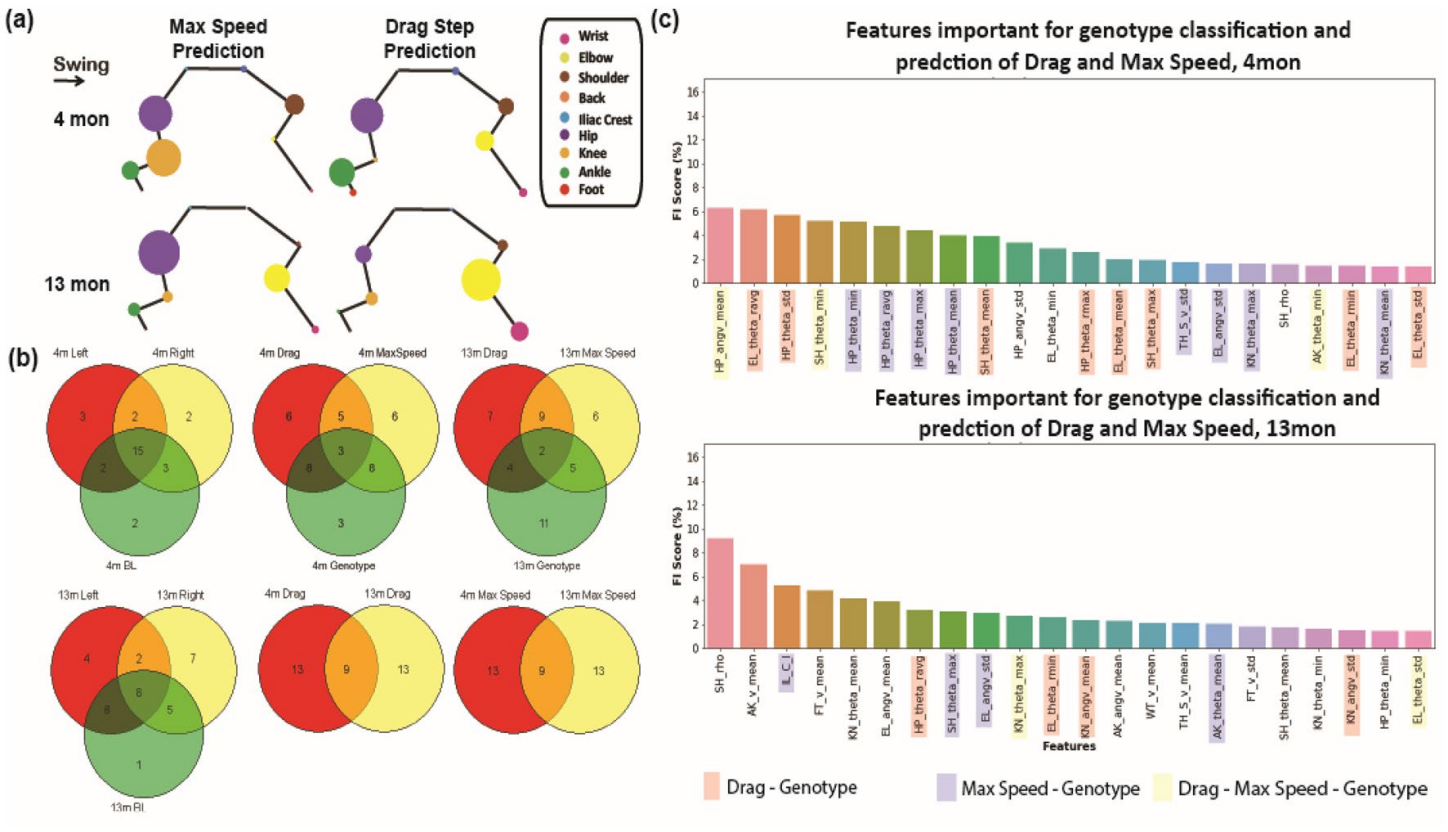
Features important for the prediction of drag steps or the maximum speed. Selected by the support vector regressor (SVR) based regression model. We investigated whether the kinematic features of treadmill stepping at 6 m/min could predict the maximum treadmill speed that the animal could run or the number of drag steps out of 10 consecutive steps. (**a**) The cumulative feature importance score (FI%) of the top 22 features for each joint for predicting the maximum speed and number of drag steps were presented for 4mon and 13mon, respectively. (**b**) The different and the common features among the 22 most important features predicting the number of drag steps or maximum speed based on SVR model, and genotype classification selected based on random forest. (**c**) The overlapping among the subsets of features optimized for genotype classification and the other two feature subsets optimized for drag step or maximum speed prediction for 4mon and 13mon. Red represents the features shared between the feature subsets optimized for drag step prediction and the genotyping classification; Blue represents the features shared between the feature subsets optimized for maximum speed prediction and the genotyping classification; Yellow represents the features shared among all three feature subsets. Panels generated through Python 3.7, MATLAB 9.2, and Adobe Illustrator (2019).

Among the different regression approaches, the SVR resulted in the lowest MAE as well as the lowest RMSE. A permutation-based approach was applied to extract the feature importance for each regression model run. For the 4mon group, the regression model yielded LOO-CV MAE of 1.38 ± 0.21 and RMSE of 1.53 ± 0.32 for the drag step and MAE of 4.80 ± 0.51 and RMSE of 4.93 ± 0.51 for the max speed. In the 13mon group, the LOO-CV mean and standard error of the MAE and RMSE were 2.71 ± 0.28 and 3.86 ± 0.19 for drag step, and 4.68 ± 0.44 and 4.98 ± 0.43 for max speed, respectively. Based on the cumulative FI score, the kinematics of hip, shoulder and hip were important for the maximum speed while the hip, elbow and ankle were most important for the drag steps in the 4mon animals. The hip and the elbow kinematics were important for both the maximum speed as well as the drag steps in the 13mon animals (Fig. 6a).

Among the 22 most important features correlated with the genotype, 11 features were also predictors of increased drag step number and decreased maximum speed in the 4mon group (Fig. 6b). In the 13mon group, the number of the features shared with the genotype classification dropped to 6 (Fig. 6b) for the drag step and 7 for the maximum speed (Supplementary Fig. 4). Furthermore, for the three features shared by all three feature subsets (Fig. 6c) in the 4mon, the ranking of feature importance for the genotype classification were: HP_angv_mean (1st), SH_theta_min (4th), and AK_theta_min (19th). For 13mon group, there are only two features shared by all three feature subsets. Neither of the two features were among the top 5 important features for the genotype classification for 13mon group (KN_theta_max, 10th; EL_theta_std, 22nd). Last but not the least, all the top 9 features important for genotype classification were also important for predicting the maximum speed, or drag step number, or both in the 4mon group. For the 13mon group, only 4 out of the top 9 important genotype classification features were also found in the prediction feature groups. The reduction in shared features classifying animals by genotype as the animal aged suggest that the decreased maximum speed and the increased number of drag steps have a closer correlation to the overexpression of hAPP in the 4mon group than in the 13mon group. It is possible that as animals age, age itself—independent of genotype—becomes a more important contributor to reduced maximum running speed and increased number of drag steps.

## Discussion

We used machine learning algorithms (Fig. 1a) to classify hAPP-overexpressing mice (J20) and control, wildtype mice into genotype classes at 4 months and 13 months based on kinematic indices of gait. The J20 animals exhibited lower maximum running speed and an increased number of dragged steps. These observations are consistent with the gait deficits reported in patients with AD. To the best of our knowledge, this is the first study to apply three-dimensional kinematic analysis to characterize a model of AD in mice. Furthermore, the classification model that we described is the first one to identify the kinematic changes specific to age and genotype-related locomotory changes. We found that machine learning algorithms detected subtle gait impairments in 4mon J20 animals, such as a change of trunk axis and imbalanced gaits, which are also observed in early AD in humans^33,42,90^. Hence, J20 mice may be used as an animal model to study gait deficits associated with AD, and a similar approach may be exploited in humans with neurological disorders.

Although we were able to classify animals into J20 or WT genotypes by measuring the number of dragged steps at 6 m/min and the highest running speed, limitations of these methods were observed. First, we had to coerce each animal to reach its maximum running speed, which caused stress and exhaustion, and similarly strenuous test conditions are not feasible in humans. Second, relying solely on previously identified indices of gait limited the exploration of novel gait features that may have diagnostic potential. Moreover, the lack of joint and muscle synergy that contributed to the decreased maximum speeds and increased the number of dragged steps in J20 mice have never been characterized.

Therefore, we used supervised machine learning algorithms without any pre-specified criteria to classify gait pattern and pathological progression in J20 and WT mice. We classified the J20 and WT mice using data gathered during locomotion at a natural, non-stressful walking speed of 6 m/min. Eighty-two kinematic features were extracted from the 3D-motion capture dataset. We used an ensemble of random forests to optimize the classification of the mice into the correct genotype within each age group based on these extracted kinematic features. We used machine learning to analyze these kinematic features without any explicit, preconceived instructions, which permitted the algorithms to detect novel kinematic features that have not been reported or recognized previously as containing potential diagnostic information. We chose impurity-based feature selection because it is more effective in terms of computational cost and modeling process efficiency, and thus more easily scalable to larger datasets, than permutation-based method for genotype classification (Supplementary Figure 2, Supplementary Table 1). With the help of supervised machine learning algorithms for classification, we were able to identify unique kinematic features for genotype classification in 4-month and 13-month animals, respectively, to quantify the importance of the kinematic features and to characterize how the gait pattern was impacted by the temporal progression of the underlying genetic abnormality.

We analyzed multiple subsets of the 82 features dataset to compare classification methods based on data from the 4mon and 13mon groups (Fig. 2). Ten features out of the 22 most important features were common among 4mon and 13mon. All 10 common features were from the elbow, shoulder, hip, and knee, indicating that the motion of these four joints was most sensitive to the hAPP-overexpression. The majority of the important features for the 4mon group (20 out of 22) were angular features (Fig. 2b), indicating that the postural changes and dyssynergia of body parts were more typical of younger J20 animals. Based on the observation of the changes of elbow and hip angles, we discovered a 2.5-degree trunk axis change in the 4-month J20 mice (Fig. 2e), which indicated the presence of postural changes early during overexpression of hAPP. We also performed the classification based on the features from the left and right side of each animal’s body in the 4mon (Fig. 3) and 13mon mice (Fig. 4). There were more common features shared by left and right in the 4mon group (17 out of 22) compared to the 13mon group (10 out 22), suggesting that the imbalance between two sides of body is more prominent after progression of the pathology caused by the overexpression of hAPP. The cumulative feature importance score (FI score) of each joint better illustrated the kinematic changes of gait patterns related to early-stage or late-stage of hAPP-induced pathologies. Differences among datasets and the accuracy of predictions based on these datasets may reflect genuine laterality in the severity of pathology in the J20 mice. However, due to the small size of the data sets and the randomized nature of the random forest algorithm, the specific ranking of the selected features may reflect random variation among the datasets and may carry little biologically meaningful information. It will be important to resolve this question with larger, denser kinematic datasets.

We applied regression models instead of classification to study the relationship between the kinematic features and maximum stepping speed and drag step number. Although the maximum stepping speed and the number of drag steps significantly correlated to the genotype as well as the age (Fig. 1), it is risky and stressful to force the animals to reach their maximum running speed or to perform too many steps with drag. Therefore, it may be possible to use the kinematic features collected at a lower, more comfortable speed (6 m/min) to predict the maximum speed and the number of drag steps. To test this hypothesis, we applied a supportive vector regression (SVR) model. Because the maximum speed and drag step number are continuous variables, the regression model better addressed our question than a dichotomous classification model. We examined the number of features shared among feature subsets selected by the two separate regression models for the maximum speed and drag steps and the subset selected by the classification model (Fig. 6b). After identifying the number of the shared features, we also checked the rank of these shared features in each genotype of mice. The greater the total number and the higher the individual ranks for the shared features are among the classification of genotype, running speed or drag step are, the stronger the correlation between the genotype and the kinematic patterns of locomotion. We identified two features with high ranks in the 4mon group and one feature with medium rank in the 13mon group. The two high ranking features, the angular speed mean of hip (HP_angv_mean, 1st) and the minimum angle of the shoulder (SH_theta_min, 4th), represent the kinematic patterns specific to the overexpression of hAPP in the 4mon J20 mice that contribute significantly to the increase of drags and the decrease of the maximum speed. Due to the much lower rank of the features shared among feature sets of the 13mon group, hAPP-overexpression seemed to make a smaller contribution to the kinematic pattern associated with the maximum speed and the number of drag steps in the older animals. Secondary effects of hAPP-overexpression, such as muscle atrophy as the animals age, could be affecting the stepping maximum speed and drags. However, one of the main limitations of this study is that we did not establishing a direct relationship between the load of hAPP in the neural system and the severity of kinematic feature patterns that we observed. Moreover, sequential measurement of time-series data from more age groups could increase the resolution of detection of gene-related progression of motor disorders^91^.

Despite the difference between the quadrupedal stepping and bipedal stepping, the patterns of hindlimb kinematics are relatively conserved due to the involvement of hind limbs body weight support, and similar analyses may be performed in humans. Based on our observations, we suggest that monitoring the speed of thigh lifting, which emerged from the shared feature HP_angv_mean and the minimum angle between the thigh and the pelvic plane (HP_theta_min), may be applicable to other transgenic models of human disorders as well as in patients with AD.

We tried to use machine learning approaches to perform a two-way classification: genotype by age. However, we were unable to obtain satisfactory accuracy in this doubly dichotomous analysis. This may reflect the relatively small size of the datasets, but equally likely, we found that the number of shared kinematic features diminished as animals aged, which corrupted the two-way classification approach—the kinematic features of 4mon and 13mon animals differed too much to allow accurate genotypic categorization across the two ages. Thus, further investigation is required to fully reveal all the kinematics features related the decrease of step speed and the increase of drag step as well as the underlying neuropathological and genetic causes of these two locomotive changes as animal age.

Gait or kinematic data acquisition is usually performed under standardized conditions on treadmills using high-speed cameras or wearable sensors or both. Kinematic datasets collected among different clinical and animal research laboratories contain similar information, and it may be possible to use machine learning to devise a quantitative gait scoring system based on previously identified standard kinematic features of gait and AD biomarkers. In principle, machine learning could be used for locomotory analysis to characterize subtle kinematic patterns of gait in multi-site and multi-center studies to compare the diagnostic accuracy of this approach in humans, which may permit earlier diagnosis or better understanding of the gait abnormalities associated with AD.

## Supplementary information

Supplementary information.

## Data Availability

The datasets generated and analyzed during the current study are available on GitHub: https://github.com/DLuLabUCLA/ML_J20_kinematics. More information are available from the corresponding author on reasonable request.
