# Supplementary material for: Machine learning classifies predictive kinematic features in a mouse model of neurodegeneration
Source: Sci Rep. 2021 Feb 17;11:3950. doi: 10.1038/s41598-021-82694-3 (PMC7889656; doi:10.1038/s41598-021-82694-3)
Supplement: Supplementary file 1 — Supplementary information. [file 41598_2021_82694_MOESM1_ESM.pdf]

Machine learning classifies predictive kinematic features in a mouse model of neurodegeneration

Authors & Affiliation: **Ruyi Huang**[1,2,3,ǂ], **Ali A Nikooyan**[1,10,ǂ], Bo Xu[1,2], M. Selvan Joseph[4], Hamidreza Ghasemi Damavandi[5], Nathan von Trotha[1,2], Lilian Li[6], Ashok Bhattarai[7], Deeba Zadeh[1], Yejie Seo[1], Xingquan Liu[1], Patrick A Truong[1], Edward H. Koo[8] , J.C. Leiter[9], Daniel C. Lu[1,2,3]*

[1]*Department of Neurosurgery, David Geffen School of Medicine, University of California, Los Angeles, Los Angeles, California 90095 USA.*

[2]*Neuromotor Recovery and Rehabilitation Center, David Geffen School of Medicine, University of California, Los Angeles, Los Angeles, California 90095 USA.*

[3]*Brain Research Institute, University of California, Los Angeles, Los Angeles, California 90095 USA.*

[4]*Department of Kinesiology, Nutritional and Food Sciences, California State University, Los Angeles, Los Angeles, California 90032 USA.*

[5]*Office of Knowledge Enterprise Development, Arizona State University, Tempe, Arizona 85281 USA.*

[6]*College of Osteopathic Medicine, Touro University Nevada, Henderson, Nevada 89014 USA.*

[7]*The Ohio State University Wexner Medical Center, Columbus, Ohio 43210 USA.*

[8]*Department of Neuroscience, San Diego School of Medicine, University of California, San Diego, La Jolla, California 92093 USA.*

[9]*Department of Molecular and Systems Biology, Geisel School of Medicine of Dartmouth College, Lebanon, New Hampshire 03756 USA.*

[10]*School of Information, University of California Berkeley, California 94720 USA.*

ǂ These Authors have equally contributed to this work and thus should be considered as joint first author
*Corresponding author:

Daniel C. Lu, M.D., Ph.D.
Associate Professor
Department of Neurosurgery
University of California, Los Angeles
300 Stein Plaza, Ste. 536
Los Angeles, CA 90095-6901
Phone: 310-267-2975
Email: dclu@mednet.ucla.edu

Supplementary Information

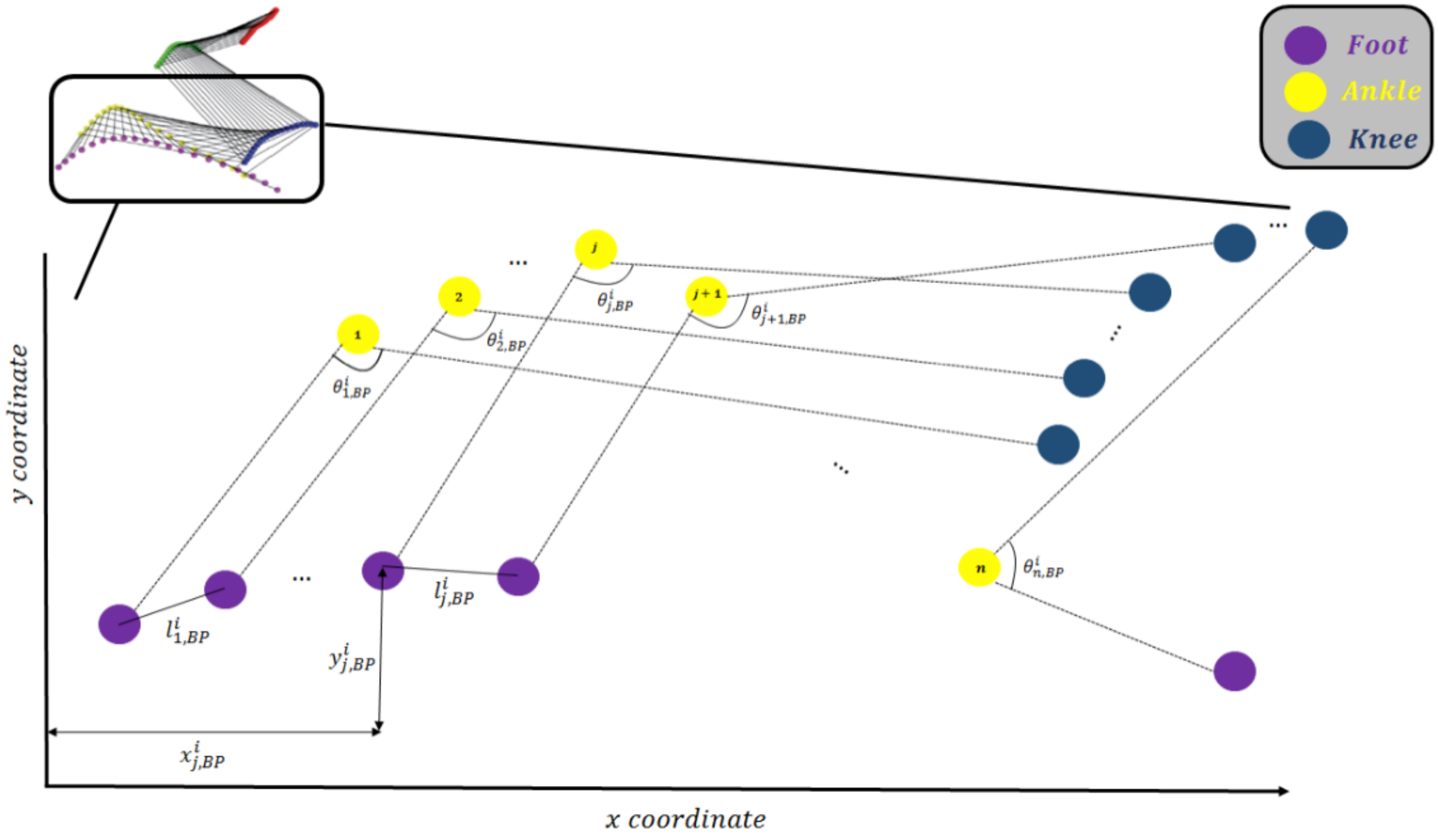

Supplementary Figure 1: An example of hindlimb trajectory and angle features extracted from five sample points within a gait cycle is shown. Panels generated through MATLAB 9.2 and Adobe Illustrator (2019).

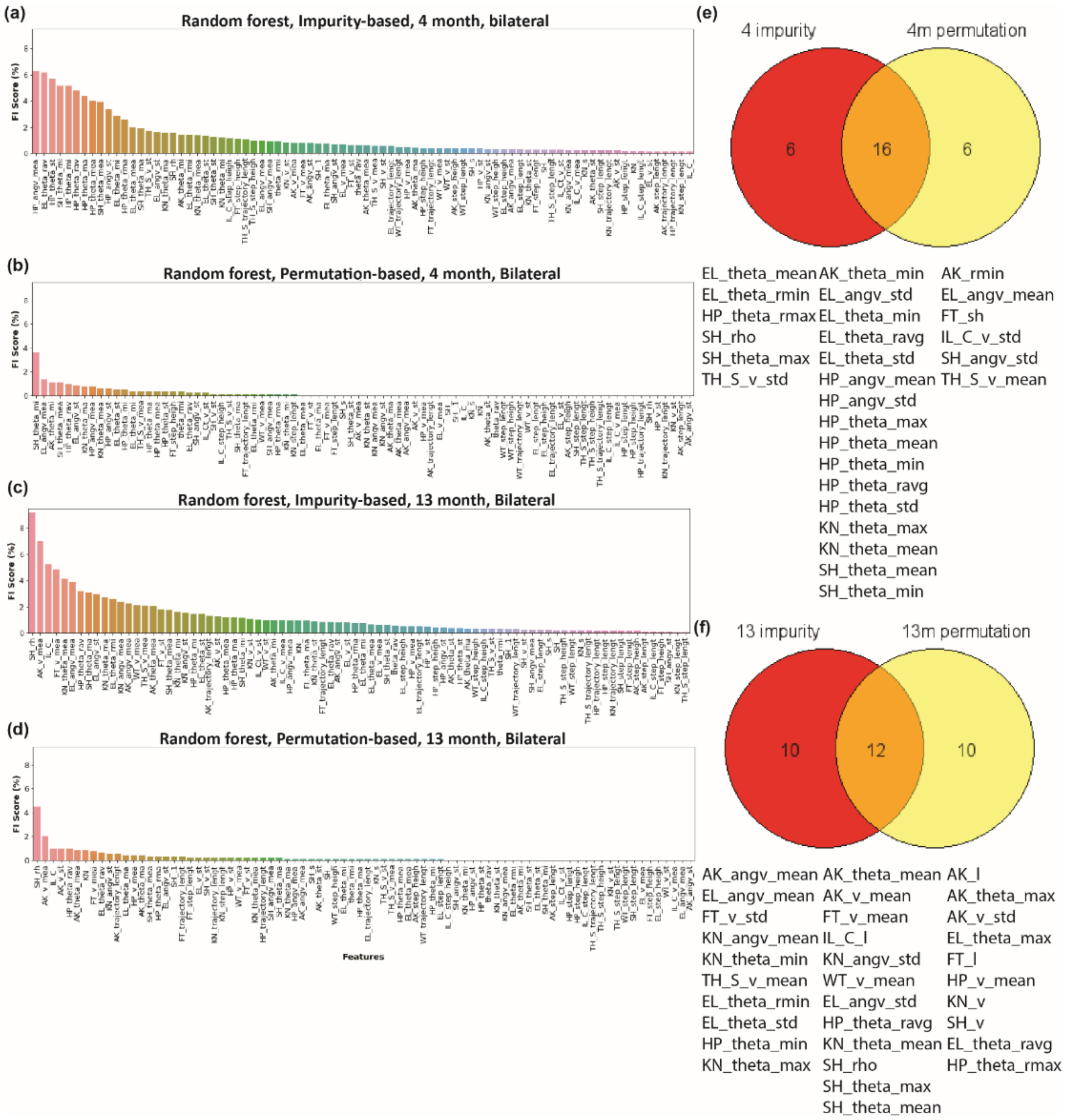

Supplementary Figure 2: Feature Importance (FI) Scores (%) calculated by the Permutation-based feature ranking of 4mon-bilateral (a) and 13mon-bilateral (c) datasets. The features ranking generated by impurity-based methods for 4mon (b) and 13mon (d) bilateral datasets. The top 22 ranked features selected by the two methods were compared for 4mon (e) and 13mon (f) group.

SH: shoulder, EL: elbow, AK: ankle, WT: wrist, FT: foot, THS: thoracic-spine, ILC: iliac-crest, HP: hip, ang: angle, v: velocity, angv: angular velocity, traj: trajectory, avg: average, std: standard deviation, l: length, max:

maximum, min: minimum, sh: step height, sl: step length. Panels generated through Python 3.7, MATLAB 9.2, and Adobe Illustrator (2019).

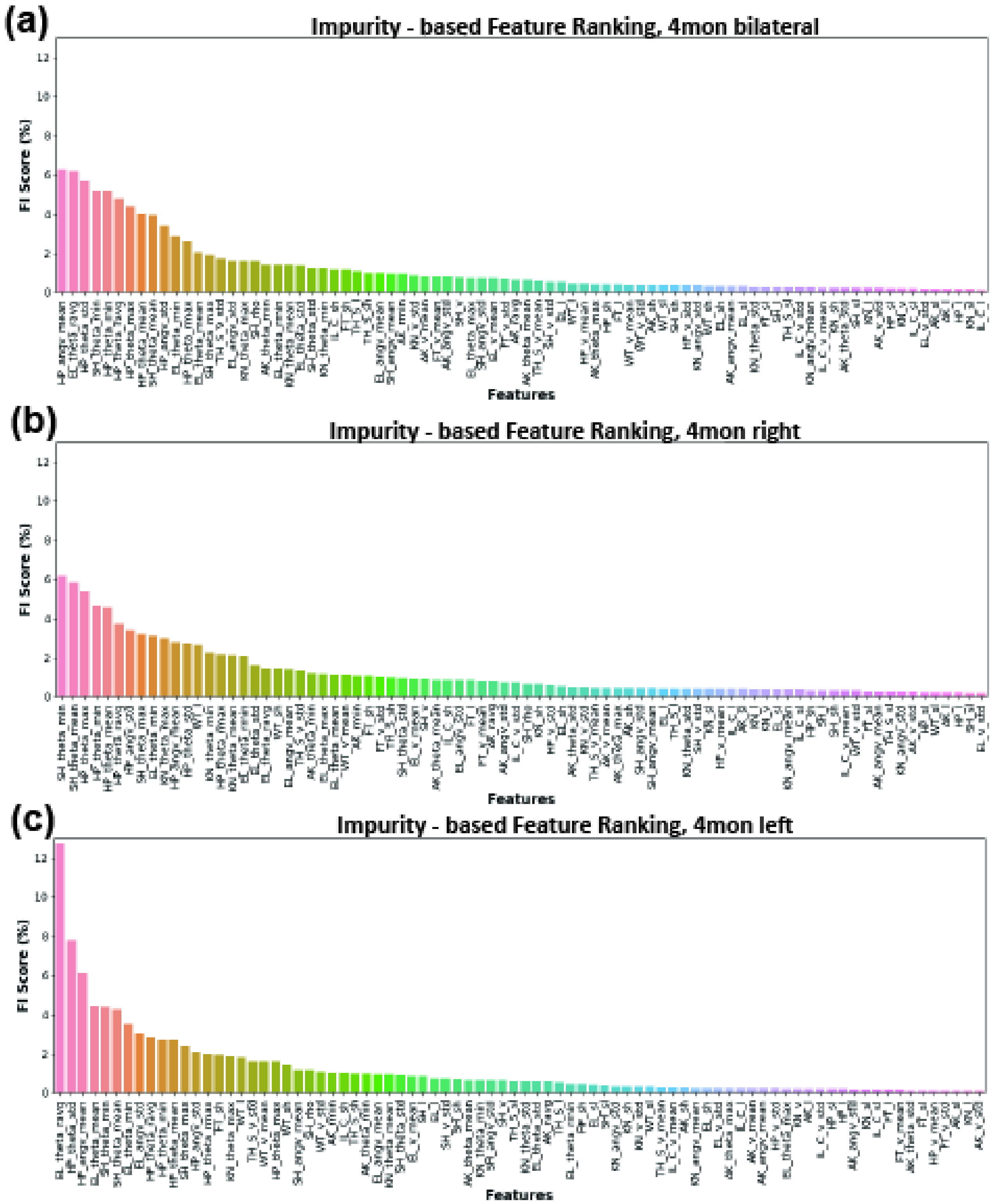

Supplementary Figure 3: Feature Importance (FI) scores (%) calculated by the Random Forest (impurity-based) feature importance method using the (a) 4monbilateral, (b) 4mon

right-side-only, (c) 4mon left-side-only datasets. All 82 observed features are shown. Key: shoulder (SH), elbow (EL), ankle (AK), wrist (WT), foot (FT), thoracic-spine (THS), iliac crest (ILC), hip (HP), angle (ang), velocity (v), angular velocity (angv), trajectory (traj), average (avg), standard deviation (std), length (len). Panels generated through Python 3.7 and Adobe Illustrator (2019).

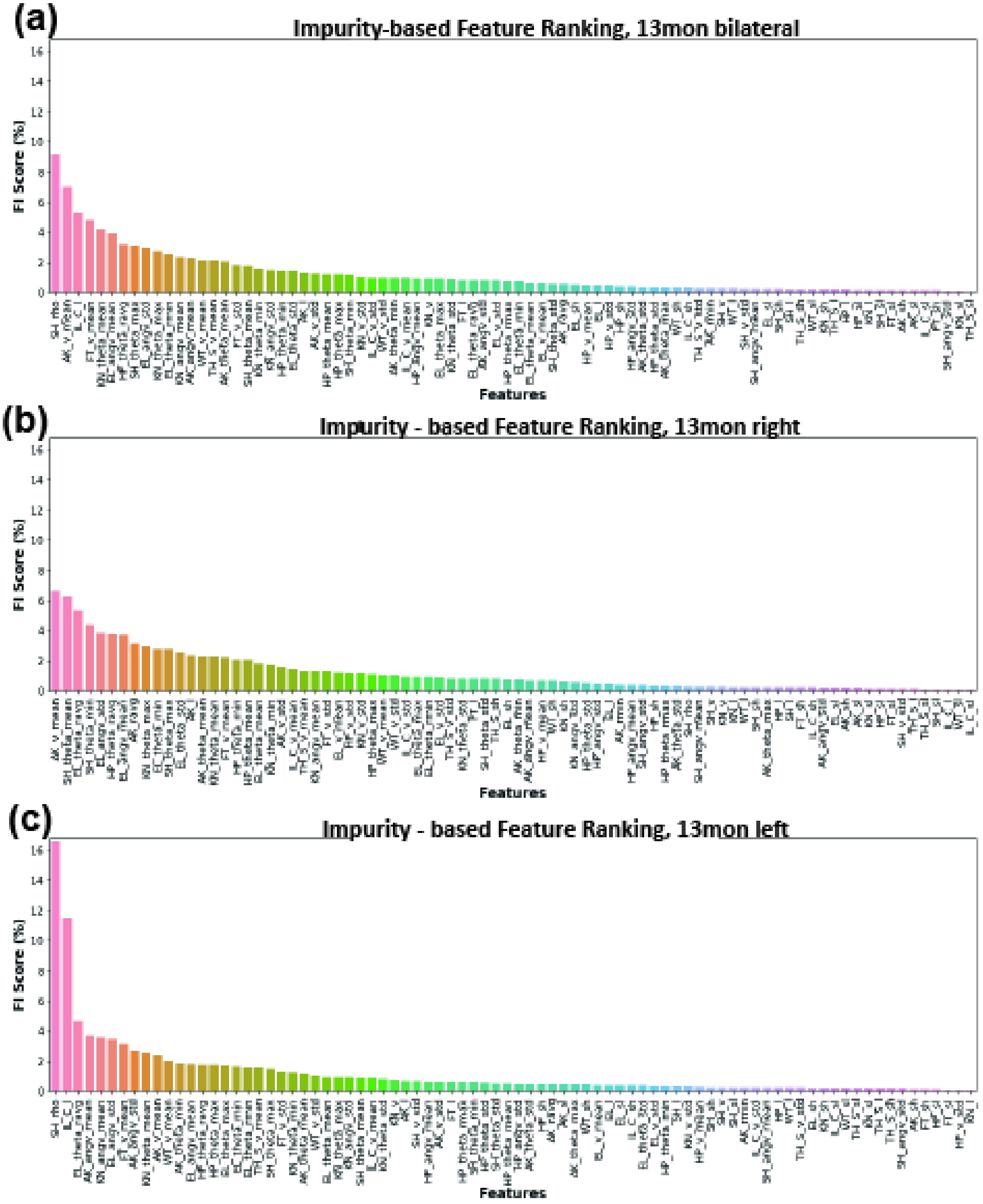

Supplementary Figure 4: Feature Importance (FI) scores (%) calculated by the Random Forest (impurity-based) feature importance method using the (a) 13mon bilateral, (b) 13mon

right-side-only, (c) 13mon left-side-only datasets. All 82 observed features are shown. Key: shoulder (SH), elbow (EL), ankle (AK), wrist (WT), foot (FT), thoracic-spine (THS), iliac crest (ILC), hip (HP), angle (ang), velocity (v), angular velocity (angv), trajectory (traj), average (avg), standard deviation (std), length (len). Panels generated through Python 3.7 and Adobe Illustrator (2019).

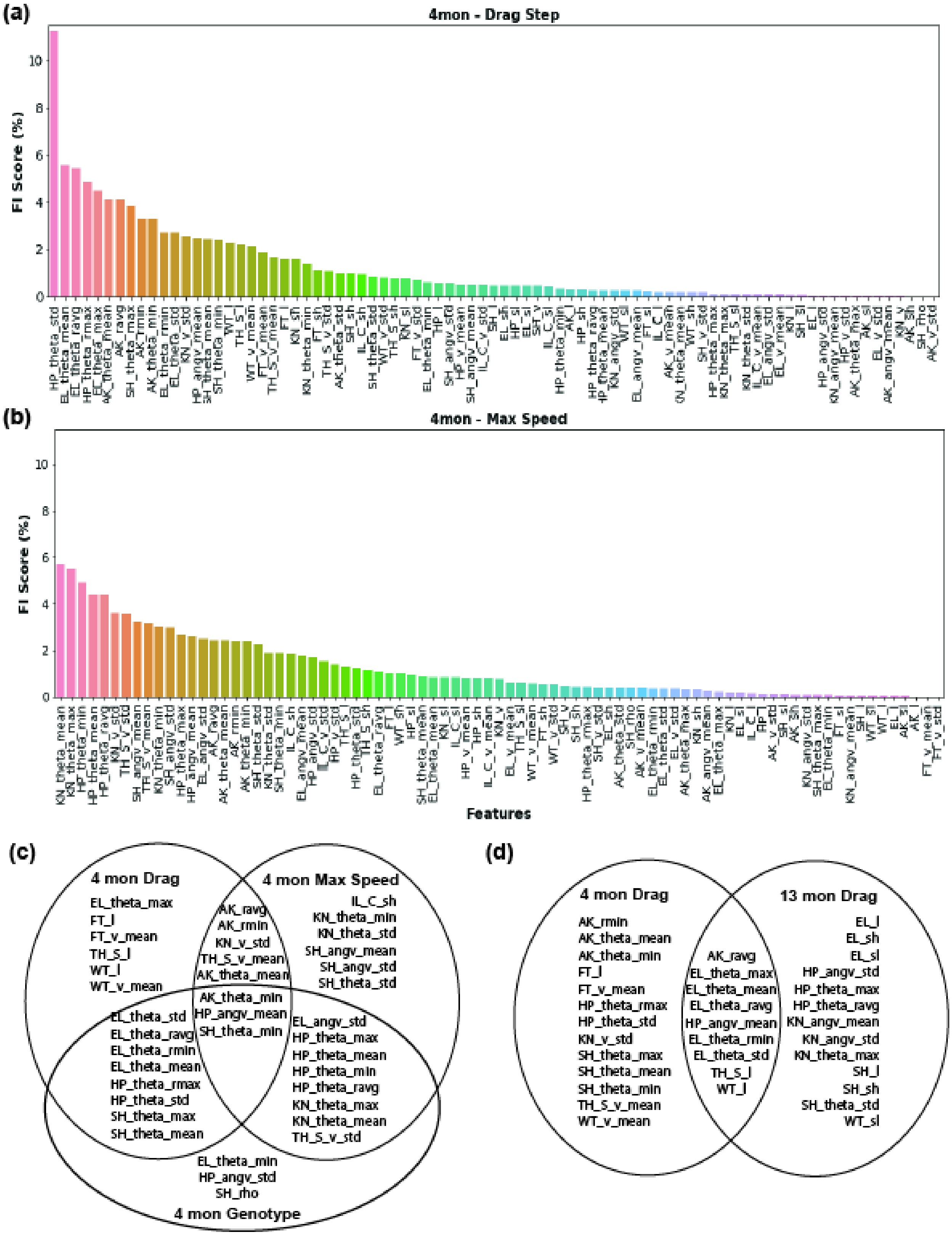
(a)
4mon - Drag Step
FI Score (%)
(b)
4mon - Max Speed
FI Score (%)
Features
(c)
4 mon Drag
EL_theta_max
FT_l
FT_v_mean
TH_S_l
WT_l
WT_v_mean
4 mon Max Speed
IL_C_sh
KN_theta_min
KN_theta_std
SH_angv_mean
SH_angv_std
SH_theta_std
AK_ravg
AK_rmin
KN_v_std
TH_S_v_mean
AK_theta_mean
AK_theta_min
HP_angv_mean
SH_theta_min
EL_theta_std
EL_theta_ravg
EL_theta_rmin
EL_theta_mean
HP_theta_rmax
HP_theta_std
SH_theta_max
SH_theta_mean
EL_angv_std
HP_theta_max
HP_theta_mean
HP_theta_min
HP_theta_ravg
KN_theta_max
KN_theta_mean
TH_S_v_std
EL_theta_min
HP_angv_std
SH_rho
4 mon Genotype
(d)
4 mon Drag
AK_rmin
AK_theta_mean
AK_theta_min
FT_l
FT_v_mean
HP_theta_rmax
HP_theta_std
KN_v_std
SH_theta_max
SH_theta_mean
SH_theta_min
TH_S_v_mean
WT_v_mean
AK_ravg
EL_theta_max
EL_theta_mean
EL_theta_ravg
HP_angv_mean
EL_theta_rmin
EL_theta_std
TH_S_l
WT_l
13 mon Drag
EL_l
EL_sh
EL_sl
HP_angv_std
HP_theta_max
HP_theta_ravg
KN_angv_mean
KN_angv_std
KN_theta_max
SH_l
SH_sh
SH_theta_std
WT_sl

Supplementary Figure 5: Feature Importance (FI) scores (%) calculated by the supportive vector machine based regression model to predict the number of drag steps and the maximum speed of animals at 13mon regardless of their genotypes. For 4mon animals, the feature ranking based on the importance of predicting the drag step number (a) or the maximum speed (b) are different. The overview of the top 22 important features for genotype classification, drag step prediction, and the maximum speed prediction with 4mon dataset (c) shows the features shared among the feature subsets as well as the differences. (d) showed the different and shared important features among 4mon and 13mon for predicting the drag step. All 82 observed features were shown in a and b. Only the top 22 features were shown in c and d. Key: shoulder (SH), elbow (EL), ankle (AK), wrist (WT), foot (FT), thoracic-spine (THS), iliac crest (ILC), hip (HP), angle (ang), velocity (v), angular velocity (angv), trajectory (traj), average (avg), standard deviation (std), length (len). Panels generated through Python 3.7 and Adobe Illustrator (2019).

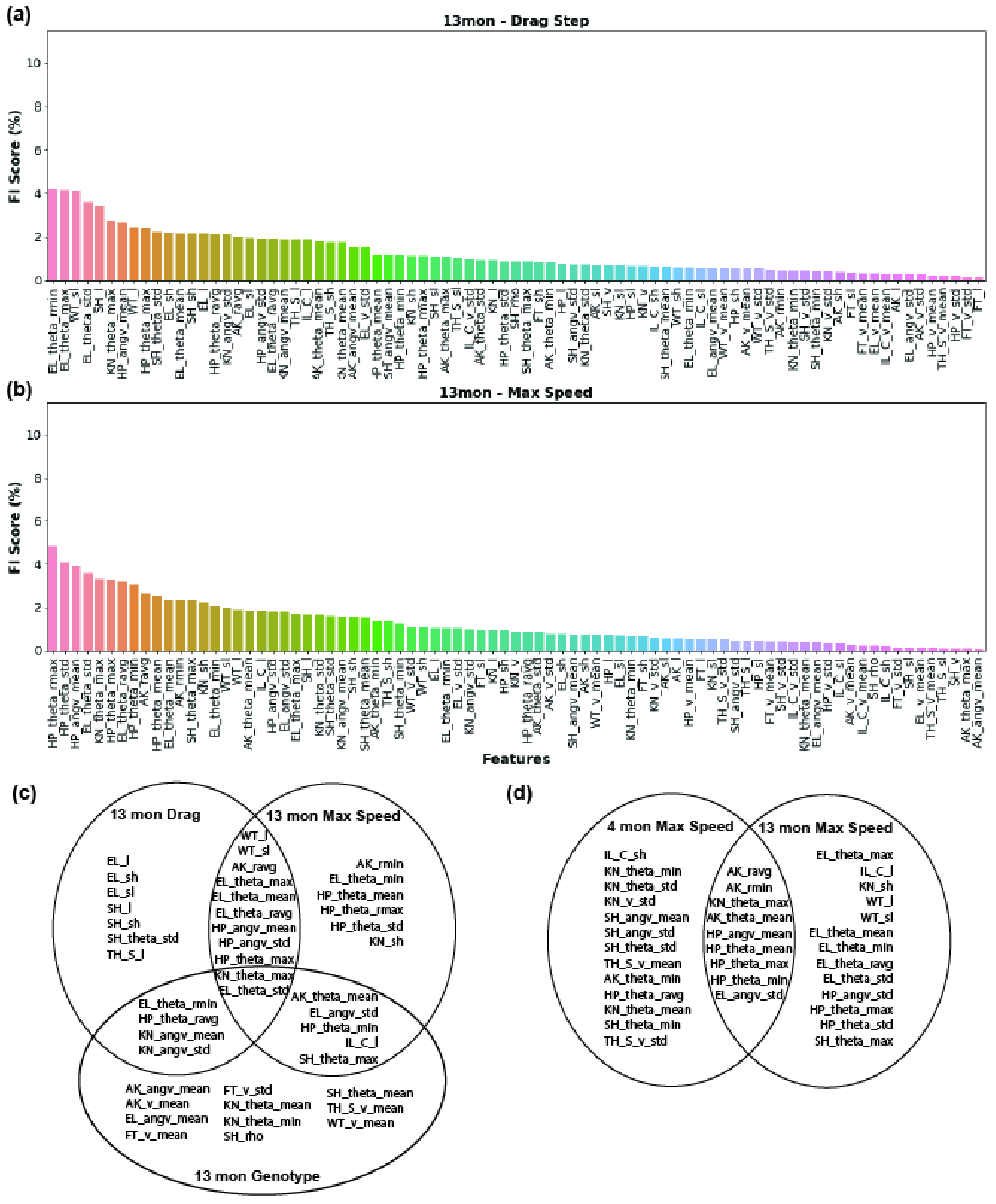

Supplementary Figure 6: Feature Importance (FI) scores (%) calculated by the supportive vector machine based regression model to predict the number of drag steps and the maximum speed of animals at 13mon regardless of their genotypes. The feature ranking based

on the importance for drag step (a) and maximum speed (b) prediction in the 13mon animals were also performed, regardless of the genotype. (c)The similar overview of the top 22 important features for 13mon animals. (d) showed the different and shared important features among 4mon and 13mon for predicting the maximum speed (h). All 82 observed features were shown in a and b. Only the top 22 features were shown in c and d. Key: shoulder (SH), elbow (EL), ankle (AK), wrist (WT), foot (FT), thoracic-spine (THS), iliac crest (ILC), hip (HP), angle (ang), velocity (v), angular velocity (angv), trajectory (traj), average (avg), standard deviation (std), length (len). Panels generated through Python 3.7 and Adobe Illustrator (2019).

Age Group	Data	LOO-CV mean b-acc. ± SEM (%)	LOO-CV mean FNR ± SEM (%)
4-mon	FI_{IMP}	84.6±6.7	0.0±0.0
	FI_{PER}	84.6±6.6	0.0±0.0
	$FI_{IMP\cap PER}$	88.5±6.1	0.0±0.0
13-mon	FI_{IMP}	93.3±4.5	0.0±0.0
	FI_{PER}	93.3±4.4	0.0±0.0
	$FI_{IMP\cap PER}$	93.3±4.5	0.0±0.0

Supplementary Table 1: The leave-one-out cross validation (LOO-CV) balanced accuracy (b-acc.) and false negative rate (FNR) mean with standard error of the mean (SEM) were used as metrics for the reduced datasets of selected features for retraining and retesting with random forest model. IMP: impurity-based feature ranking, PER: permutation-based feature ranking,

	4mon BL	4mon R	4mon L	13mon BL	13mon R	13mon L
Wrist	0.00	4.08	4.82	2.11	0	2.2
Elbow	15.42	9.65	23.67	10.81	17.60	14.88
Shoulder	12.60	15.24	12.17	13.99	13.42	18.04
Thoracic Spine	1.71	1.32	1.58	2.08	0	1.59
Iliac Crest	0.00	0.00	0.00	5.26	1.44	11.48
Hip	36.26	29.30	27.77	4.59	7.91	3.57
Knee	2.99	7.33	1.86	12.38	6.91	7.35
Ankle	1.42	0.00	0.00	11.33	16.01	10.61
Foot	0.00	0.00	1.93	6.61	2.22	4.45

Supplementary Table 2. The cumulative FI scores (%) of nine anatomical locations were calculated for six datasets. The color codes indicate the cumulative FI: grey: <5%, green: 5< <10%, yellow: 10< <20%, red: >20%.
